# Motor hyperactivity of the iron‐deficient rat — an animal model of restless legs syndrome

**DOI:** 10.1002/mds.27133

**Published:** 2017-08-26

**Authors:** Yuan‐Yang Lai, Yu‐Hsuan Cheng, Kung‐Chiao Hsieh, Darian Nguyen, Keng‐Tee Chew, Lalini Ramanathan, Jerome M. Siegel

**Affiliations:** ^1^ Department of Psychiatry and Biobehavioral Sciences University of California, Los Angeles Los Angeles California USA; ^2^ Veterans Administration Greater Los Angeles HealthCare System Sepulveda, Los Angeles Los Angeles California USA

**Keywords:** sleep, hematocrit, periodic leg movement

## Abstract

**Background:**

Abnormal striatal dopamine transmission has been hypothesized to cause restless legs syndrome. Dopaminergic drugs are commonly used to treat restless legs syndrome. However, they cause adverse effects with long‐term use. An animal model would allow the systematic testing of potential therapeutic drugs. A high prevalence of restless legs syndrome has been reported in iron‐deficient anemic patients. We hypothesized that the iron‐deficient animal would exhibit signs similar to those in restless legs syndrome patients.

**Methods:**

After baseline polysomnographic recordings, iron‐deficient rats received pramipexole injection. Then, iron‐deficient rats were fed a standard rodent diet, and polysomnographic recording were performed for 2 days each week for 4 weeks.

**Results:**

Iron‐deficient rats have low hematocrit levels and show signs of restless legs syndrome: sleep fragmentation and periodic leg movements in wake and in slow‐wave sleep. Iron‐deficient rats had a positive response to pramipexole treatment. After the iron‐deficient rats were fed the standard rodent diet, hematocrit returned to normal levels, and sleep quality improved, with increased average duration of wake and slow‐wave sleep episodes. Periodic leg movements decreased during both waking and sleep. Hematocrit levels positively correlated with the average duration of episodes in wake and in slow‐wave sleep and negatively correlated with periodic leg movements in wake and in sleep. Western blot analysis showed that striatal dopamine transporter levels were higher in iron‐deficient rats.

**Conclusions:**

The iron‐deficient rat is a useful animal model of iron‐deficient anemic restless legs syndrome. © 2017 The Authors. Movement Disorders published by Wiley Periodicals, Inc. on behalf of International Parkinson and Movement Disorder Society

Restless legs syndrome (RLS) is a sensory‐motor disorder^1^, ^2^ characterized by unpleasant and painful sensations and is usually accompanied by paresthesias. These uncomfortable sensations occur during rest or while lying down, typically appear in the late afternoon or evening, and are relieved by movement. Thus, RLS patients suffer from the inability to fall asleep and stay asleep, resulting in profound insomnia.^3^ Restless legs syndrome has been reported in patients with iron‐deficient (ID) anemia,^4^ Parkinson's disease,^5^, ^6^ and end‐stage renal disease,^7^ as well as in children with attention deficit hyperactivity disorder^8^ and in pregnant women.^9^ Despite RLS disorder occurring in all age groups and affecting 5%‐10% of the population, making it one of the most common movement disorders,^10^ there is no satisfactory treatment and no suitable animal model that would allow the development of better treatments.

The subjective abnormal sensations have made it difficult to develop an animal model of RLS. However, periodic leg movements (PLMs), an objective motor event, can be recorded in animals. PLMs in quiet wake is common in RLS patients.^11^, ^12^ More than 85% of RLS patients also have PLMs in sleep.^13^ Clinical studies have shown a high prevalence of RLS in ID anemic patients.^14^ Neural abnormalities in the substantia nigra and abnormal sleep architecture have been reported in ID rats and mice, respectively.^15^, ^16^ However, motor activity in quiet wake and in sleep, a key element of restless legs syndrome, has not been studied in ID animals. In this study, we examine whether ID rats have abnormal sleep patterns and motor activity, using video and polysomnographic recording techniques. Abnormal striatal dopamine transmission — increased, decreased, or unchanged dopamine transporter (DAT) level — has also been reported in idiopathic RLS patients.^17^, ^18^, ^19^ Thus, we have measured striatal DAT levels in ID rats compared with control rats, using the Western blot technique.

## Methods

All procedures were approved by the Institutional Animal Care and Use Committee of the VA Greater Los Angeles Healthcare System.

### Development of Iron‐Deficient Rat

Thirteen Sprague‐Dawley weanling rats (21 days old) were purchased from Charles River Laboratory and then divided into 2 groups: 6 normal controls and 7 iron‐deficient (ID) rats. Control rats were fed with standard rodent diet containing 35 ppm iron, whereas ID rats were fed with a rodent diet containing 4 ppm iron (TD.80396; Harlan Teklad Lab) for 2 months. All rats were then surgically implanted with electrodes for sleep and motor activity recordings.

### Surgery

Implantation of electrodes for electroencephalograph (EEG) and electromyograph (EMG) recordings in the animal was described in our previous study.^20^ Briefly, under isoflurane (1.5%) anesthesia, 3 jeweler's screw electrodes were implanted over the cortex (A, anterior to lambda; L, lateral from the midline; A14, L2.5; A10, L 3.5; and A5, L1) for cortical EEG recording. Flexible multistranded stainless steel wires (793500; A‐M Systems, Inc., Carlsborg, WA) were inserted into the nuchal and hindlimb musculature bilaterally and routed subcutaneously to the skull for EMG recording. Wires from all electrodes were soldered to a 14‐pin Amphenol strip connector and encased in an acrylic head plug.

### Polysomnographic and Video Recordings

Animals were allowed to recover from surgery for a week. Animals were individually housed in sound‐attenuated chambers with a 12:12 light‐dark cycle and ad libitum access to food and water. Electrophysiological signals were collected and amplified through a polygraph (model 15LT; Grass, MA) and then digitized and recorded via a Micro 1401 interface (Cambridge Electronics Design, Cambridge, UK). Sleep and phasic leg movements were visually scored offline with a tailored script in Spike2 (Cambridge Electronics Design, Cambridge, UK). Infrared cameras were used for video recordings. Video images were captured digitally through a 4‐channel surveillance video recorder card (Q‐See QSPDVR04; RapidOS, New Taipei City, Taiwan). Video was recorded continuously with time stamps matched to polysomnographic recordings.

### Pramipexole Injection

After 3 days of baseline polysomnographic recordings, 5 each control and ID rats were systemically (intraperitoneally) administered saline or 3 doses (0.01, 0.02, and 0.05 mg/kg) each of pramipexole at Zeitgeber (ZT) 2. Sleep and motor activity recordings were continued for 8 hours after saline or test drug injection.

### Iron‐Replacement Experiment

After saline and test drug injection experiments, ID rats were fed standard rodent diet containing 35 ppm iron for up to 4 weeks. Sleep and motor activity recordings resumed on day 6 post–standard rodent diet feeding. Sleep recordings were performed for 2 days continuously each week, for an additional 3 weeks.

### Hematocrit Measurement

Blood was collected from the lateral tail vein, transferred into a heparinized hematocrit capillary, and centrifuged at 10,000 rpm for 3 minutes in a hematocrit centrifuge (BD Clay Adams 420563). Hematocrit levels were calculated by measuring the length of the red blood cell layer against the total blood layer.

At the end of the experiment, all rats were deeply anesthetized with isoflurane and their brains removed. The striatum was dissected and stored at ‐80°C.

### Western Blot Experiment

The striatum was separately sonicated in lysis buffer (50 mM Tris, 5 mM EDTA, 30% igepal NP‐40, 10% Na deoxycholate, and 1% sodium dodecyl sulfate [pH 7.5]) and protease inhibitor tablet (Roche 4693124) and then centrifuged at 10,000*g* for 20 minutes at 4°C. Protein concentration of the supernatant was determined using a DC protein assay kit (BioRad 500‐0112) and read at 650 nm in an E_max_ Precision microplate reader (Molecular Devices) using a Softmax program. Ten micrograms of striatal protein was loaded on a 10% mini‐protean TGX precast gel (Biorad 456‐1034) and electrophoresed for 1.5 hours at room temperature at 120 V. Proteins were then transferred to a polyvinylidene fluoride membrane for 1.5 hours at 100 mA at room temperature. Membranes were washed in Tris‐buffered saline with tween (TBST: 20 mM Tris, 150 mM NaCl, 0.1% tween) and then blocked in TBST containing 5% nonfat dry milk for 1 hour at room temperature. Membranes were subsequently incubated with rabbit anti‐DAT (1:2,000; Millipore AB2231), and mouse antiactin (1:10,000; Millipore MAB1501R) overnight at 4°C, goat antirabbit IgG conjugated with horseradish peroxidase (HRP; 1:10,000; Jackson Immunores Lab 111‐035‐144) and goat antimouse IgG conjugated with HRP (1:10,000, Jackson Immunores Lab, 715‐035‐140) for 1 hour and West Femto (1:80; Thermo Scientific 34094) for 5 minutes. Membranes were washed 3 times of TBST between incubations. Dopamine transporter (75 KDa) and actin (43 KDa) were detected and visualized using a ChemiDoc XRS+ system (Biorad). Striatal proteins obtained from the normal control, ID, iron replacement week 1 (IR1), week 2 (IR2), week 3 (IR3), and week 4 (IR4) rats were analyzed simultaneously on the same gels and blots.

### Data Analysis

The CED 1401 Spike 2 program was used to analyze EEG power spectra, as well as to detect and score phasic motor activity during quiet wake and sleep. Periodic leg movements in quiet wake (PLMW) and in slow‐wave sleep (SWS; or nonrapid eye movement sleep) (PLMS) were analyzed by adapting the criteria outlined by the World Association of Sleep Medicine standards for scoring PLM.^21^ In brief, phasic motor events (jerks) in the leg, satisfying the following criteria, were counted as PLMs: (1) the amplitude of the jerk was twice the tonic background; (2) the duration of the jerk ranged between 0.2 and 5 second; (3) the interval between jerks was less than 90 seconds; and (4) at least 4 consecutive jerks fulfilled criteria 1‐3. Phasic motor events, which did not meet criterion 4 of PLM in SWS, were counted as isolated leg movements in sleep (ILMS). The index of PLM in quiet wake (PLMWI) and in SWS (PLMSI) was calculated as the total number of periodic motor movements divided by total time in quiet wake or in SWS, respectively. Similarly, the index of ILMS (ILMSI) was calculated as the total number of ILMS divided by total time in sleep.

To determine the density of dopamine transporter in the striatum, the optical density of the dopamine transporter band was measured using Quantity One 1‐D analysis software (Biorad). Relative levels of striatal dopamine transporter in each animal were obtained by measuring the ratio of the optical density of dopamine transporter and actin.

One‐way ANOVA with replicated measures followed by a Bonferroni's *post hoc, t*‐test and Pearson correlation test were used for statistical analysis.

## Results

### Hematocrit Levels, Sleep Patterns, and Motor Activity in Control and Iron‐Deficient Rats

Average hematocrit level of normal rats was 47.1 ± 0.2, whereas hematocrit levels of ID rats were significantly lower, at 20.6 ± 3.2 (*P* < 0.001, *df* = 11, *t* test; Table 1).

**Table 1:** Hematocrit levels, percent of wake‐sleep stages, average duration (minutes) of sleep‐wake episodes, and motor activity in normal control, iron‐deficient, and iron‐replacement rats

	Hct	Percent of sleep‐wake stages^a^			Average duration of episode^a^			Motor activity^a^		
		Wake^b^	SWS^b^	REM^b^	Wake^b^	SWS^b^	REM^b^	PLMSI	PLMWI	ILMSI
C	47.1 ± 0.1^c^	49.5 ± 2	44.5 ± 1.7	6 ± 0.9	1.95 ± 0.35^a^	1.67 ± 0.31^c^	1.1 ± 0.13	7.8 ± 2.6^c^	0^c^	18.8 ± 5.5
ID	25.2 ± 1.5^c^, ^d^	49.7 ± 3.9	45.6 ± 3.2	4.7 ± 1^c^, ^d^	1.41 ± 0.13^c^, ^d^	1.28 ± 0.21^c^, ^d^	1.21 ± 0.27	49.8 ± 16.9^c^, ^d^	14 ± 29.4^c^	16.9 ± 2.2
IR1	38.7 ± 1.1^d^, ^e^	47.7 ± 1.3	46.1 ± 1	6.2 ± 0.9	1.97 ± 0.17^d^	1.79 ± 0.2^d^	1.27 ± 0.17	18.5 ± 6.9^d^, ^e^	5.8 ± 6.9	18.6 ± 3.9
IR2	41 ± 0.6^d^, ^e^	46.3 ± 2.9^e^	47.3 ± 2.2^e^	6.4 ± 1^d^	1.95 ± 0.13^d^	1.87 ± 0.18^d^	1.31 ± 0.23	9.3 ± 6.4^d^	2.7 ± 3.8	17.6 ± 7.7
IR3	44.8 ± 0.3^d^, ^e^	46 ± 4^e^	47.9 ± 3.5^e^	6.1 ± 1	2.02 ± 0.3^d^	1.98 ± 0.24^d^	1.22 ± 0.21	6.8 ± 4^d^	1.4 ± 1.7	18.5 ± 5.3
IR4	47.2 ± 0.1^d^, ^e^	46.2 ± 3.2^e^	47.9 ± 2.8^e^	5.9 ± 0.8	1.98 ± 0.2^d^	1.96 ± 0.24^d^	1.23 ± 0.23	4.6 ± 4.6^d^	1 ± 1.5	18 ± 6.2

Number of animals — control, 6; ID and IR1‐IR4, 7 each.

Hct, hematocrit level; W, wake; SWS, slow‐wave sleep; REM, rapid eye movement sleep; PLMSI and PLMWI, index of periodic leg movements in sleep and in quiet wake, respectively; ILMSI, index of isolated leg movements in sleep; C, normal control rat; ID, iron‐deficient rat; IR1‐IR4: ,iron replacement (IR) rats fed with standard rodent diet for 1, 2, 3, or 4 weeks.

a Data (mean ± SD) taken from 24‐hour recording.

b Minutes.

c Significant difference between ID and control rats.

d Significant difference between ID and IR rats.

e Significant difference between IR and control rats;.

Neither the percentage of SWS (*P* > 0.05, *df* = 11, *t*‐test; Table 1), nor the percentage of wake (*P* > 0.05, *df* = 11, *t* test; Table 1) in ID rats was different from control rats. In contrast, the percentage of REM sleep was lower in ID rats than in control rats (*P* < 0.05, *df* = 11, *t* test; Table 1). Sleep fragmentation was observed in ID rats. Statistical analysis showed that the daily average duration of a waking episode (*P* < 0.01, *df* = 11, *t* test; Table 1) and an SWS episode (*P* < 0.05, *df* = 11, *t* test; Table 1) was shorter in ID rats than in control rats.

**Figure 1:**
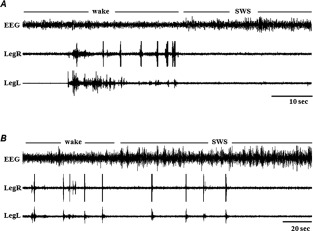
Periodic leg movements in the right (A) or both the right and left (B) hindlimbs, in wake, before the animal falls asleep. Periodic leg movements were not observed in sleep (A), whereas PLM was gradually diminished in sleep (B). LegL and LegR, left and right hindlimb EMG.

Motor hyperactivity in wake and in SWS was observed in the ID rats. Periodic leg movements in wake, which were not seen in control rats and in 2 ID rats, were observed in 5 ID rats. Periodic leg movements in wake were often seen immediately before the animal fell asleep (Fig. 1). A very high amount of PLM in sleep (see the video) was seen in ID rats. PLM occurred in one (Fig. 1A) or both (Fig. 1B) hindlimbs in all ID rats. The PLMWI (*P* < 0.001, *df* = 11, *t* test; Table 1) and PLMSI (*P* < 0.001, *df* = 11, *t* test; Table 1) in ID rats were significantly higher than in normal rats. PLMWI and PLMSI were significant higher in ID rats than in control rats in both the light phase (PLMWI: *df* = 11, *t* test, *P* < 0.001; PLMSI: *df* = 11, *t* test, *P* < 0.001) and dark phase (PLMWI: *df* = 11, *t* test, *P* < 0.001; PLMSI: *df* = 11, *t* test, *P* < 0.001). However, isolated leg movements in sleep did not differ between control and ID rats (*df* = 11, *t* test, *P* = 0.19; Table 1).

### Effect of Pramipexole Treatment on Sleep and Motor Activity in ID Rats

Intraperitoneal injection of pramipexole, a dopamine D_3_ agonist and commonly used for the treatment of RLS, decreased PLM in sleep (*df* = 4, ANOVA; *P* < 0.001) during 8 hours of recording after injection (Table 2) in ID rats. Post hoc analysis showed that pramipexole dose dependently decreased PLM in sleep. Although PLM in wake was decreased by pramipexole injection, the change in PLM in wake was not significant (Table 2). The sleep‐wake pattern was not changed by any dose of pramipexole injection (Table 2).

**Table 2:** Effect of intraperitoneal injection of saline or pramipexole on sleep and motor activity^a^

		Sleep‐wake states (minutes)			Motor activity		
		Wake^b^	SWS^b^	REM^b^	PLMSI	PLMWI	ILMSI
Baseline		176.7 ± 24.4	274.6 ± 21.5	28.6 ± 8.3	55.5 ± 18.8	12.8 ± 17.4	14.3 ± 3.5
Saline		167.6 ± 10.8	279.5 ± 13.9	32.9 ± 5.7	50.2 ± 15.8	19.3 ± 17.7	14.9 ± 3.5
Pramipexole (mg/kg)	0.01	166.9 ± 40.8	284.3 ± 38.6	28.8 ± 8	25.6 ± 5.6^c^, ^d^, ^e^	14 ± 20.9	20.2 ± 2.7^c^
	0.02	194.3 ± 38.4	259 ± 28.7	26.7 ± 12.9	18.2 ± 4.1^c^, ^d^, ^e^, ^f^	3.7 ± 2.9	19 ± 3.6^c^
	0.05	200.9 ± 63.3	249.2 ± 56.4	29.9 ± 9.6	14.3 ± 4.2^c^, ^d^, ^e^	1.8 ± 1.5	22.1 ± 6.1

a Data (mean ± SD) taken from the average of 8‐hour recordings after saline or drug injection at ZT2. n = 5 each, post hoc test.

b Minutes/8 hours.

c Baseline versus drug injection, *P* < 0.05.

d Saline versus drug injection, *P* < 0.05.

e Pramipexole 0.01 versus 0.02 or 0.05 mg/kg, *P* < 0.05.

f Pramipexole 0.01 versus 0.02 mg/kg.

### Hematocrit Level, Sleep Pattern, and Motor Activity in Iron‐Deficient Rat After Iron Therapy

Hematocrit level, sleep quality, and motor activity were dramatically changed starting during the first week of iron replacement. After 4 weeks of iron replacement, ID rats showed a significant increase in hematocrit level (ID vs IR1, IR2, IR3, and IR4, post hoc test, *P* < 0.001; Table 1) and a significant increase in REM sleep (*df* = 4, ANOVA, *P* = 0.001; Table 1). Although wake (*df* = 4, ANOVA, *P* = 0.08; Table 1) and SWS (*df* = 4, ANOVA, *P* = 0.25; Table 1) times were not changed, the average duration of episodes in wake (ID vs IR1, IR2, IR3, and IR4, post hoc test, *P* < 0.001; Table 1) and in SWS (ID vs IR1, IR2, IR3, and IR4, post hoc test, *P* < 0.001; Table 1) was significantly increased after iron replacement. Duration of bouts of both wake and SWS in ID rats reached control levels in week 1 post–iron replacement (Table 1). Furthermore, both the average bout duration in wake (*R* ^2^ = 0.91, n = 32, Pearson correlation test, *P* < 0.01) and in SWS (*R* ^2^ = 0.77, n = 32, Pearson correlation test, *P* < 0.05) were positively correlated with hematocrit level.

Motor hyperactivity that was seen in ID rats was also improved after iron replacement. Periodic leg movements in SWS (ID vs IR1, IR2, IR3, and IR4, post hoc test, *P* < 0.01; Table 1) were significantly decreased after the ID rat was fed standard rodent diet compared with ID rats on a low iron diet. The occurrence of PLM in sleep was reduced to control levels in week 2 post–iron replacement (Table 1). Although PLM in wake showed no change in 24 hours (*df* = 4, ANOVA, *P* = 0.14), a significant decrease was observed in the light phase (*df* = 4, ANOVA, *P* < 0.05) after iron replacement. We also found that hematocrit levels were negatively correlated with PLM in sleep (*R* ^2^ = −0.99, n = 32, Pearson correlation test, *P* < 0.001) and in wake (*R* ^2^ = −0.99, n = 32, Pearson correlation test, *P* < 0.001). In contrast, the ILMSI in ID rats was not changed after iron replacement (*df* = 4, ANOVA, *P* = 0.77).

### Striatal Dopamine Transporter Levels in Control, Iron‐Deficient, and Iron‐Replacement Rats

**Figure 2:**
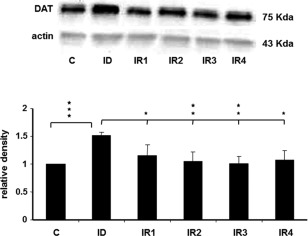
Striatal dopamine transporter (DAT) in the control (C), iron‐deficient (ID), and iron‐replacement week 1 (IR1), week 2 (IR2), week 3 (IR3), and week 4 (IR4) rats. Top: example of Western blot showing striatal DAT and actin expression in samples taken from control, ID and IRs rats. Lower: relative density of bands expressed as the ratio of DAT and actin in each animal groups. *P < 0.05, *t* test; **P < 0.01, *t* test. n = 5 each group.

Striatal dopamine transporter levels were higher in the ID rat than the control and IR rats (Fig. 2). The increased striatal dopamine transporter levels were decreased to the control level 1 week after the ID rat was fed a standard rodent diet (Fig. 2). Striatal dopamine transporter levels were negatively correlated with hematocrit levels (*R* ^2^ = ‐0.98, n = 32, Pearson correlation test, *P* < 0.001) and positively correlated with PLM in sleep (*R* ^2^ = 0.98, n = 32, Pearson correlation test, *P* < 0.001) and in wake (*R* ^2^ = 0.99, n = 32, Pearson correlation test, *P* < 0.01) in a 24‐hour recording.

## Discussion

We found that ID rats exhibit sleep fragmentation, motor hyperactivity, PLM in quiet wake, and an increase in PLM in sleep, signs resembling those in human RLS patients. We also found that iron therapy improved PLM in quiet wake and in sleep, hematocrit levels, and sleep quality. Furthermore, we found that hematocrit levels are negatively correlated with PLM in wake and in sleep, as well as positively correlated with average wake and SWS bout duration. Western blot studies showed that striatal dopamine transporter levels were higher in ID rats than in normal control and iron‐replacement rats, suggesting abnormal striatal dopamine transmission may contribute to motor hyperactivity in ID rats. The ID rat had a positive response to pramipexole treatment, indicating the ID rat is a useful animal model of ID anemic‐induced RLS.

Using polygraphic recording simultaneously with real‐time video recording, we were able to observe and record behavioral activities. An increase in PLM in sleep was observed in all ID rats. Montplaisir et al^13^ reported that 80% of RLS patients had PLM in sleep. The difference in RLS patients and ID rats in PLM in sleep may be because of the unique factor (low hematocrit level) in ID rats. However, PLM in wake was not observed in 2 of 7 ID rats. Montplaisir et al reported that 20% of RLS patients did not show PLM in the immobilization test.^22^ The ID rat without PLMs in wake may not have abnormal sensations and/or urge to move; however, they have PLM disorder.

Clinical studies have shown inconsistent responses of motor activity to iron therapy in RLS patients, with a decrease in PLM in sleep^23^, ^24^ or no change in PLM in sleep or in wake.^25^ In our study, we found that PLM in sleep decreased after ID animals are fed a standard rodent diet, which contains 35 ppm iron. The decrease in PLM and increase in hematocrit levels were most profound after the first week of iron replacement. Although PLM in wake was also decreased after the iron therapy, the decrease in PLM in wake was not significant. This may be because of the high variation of the baseline, which included data from 2 ID rats without PLM in wake. Isolated leg movements in sleep were not affected by iron therapy. However, a significant decrease in PLM in wake was found in the light phase after iron therapy, as well as during pramipexole treatment (Table 2) in the light phase, indicating the animal awakening from sleep less frequently.

In the present study, we found that iron deficiency decreases REM sleep and causes sleep fragmentation and a shorter duration of wake and SWS episodes. These sleep disorders can be reversed by iron replacement. Decreased PLM in sleep and increased SWS bout duration indicate that iron‐replacement animals awaken less often, resulting in more consolidated sleep. Dean et al^15^ reported that total sleep time over 24 hours and during the light and dark periods did not differ between ID and normal mice. However, they did observe significantly increased wake and significantly decreased SWS during the last 4 hours of the dark phase in ID mice.^15^ We did not observe changes in total amount of sleep or wake time in ID compared with normal rats in any period, including the last 4 hours of the dark phase (data are not shown). There may be a species difference in altered sleep architecture.

Eisensehr et al^17^ and Conner et al^16^ reported that the concentration of the striatal dopamine transporter did not differ between drug‐naive and levodopa‐treated idiopathic RLS patients and controls. On the other hand, Earley et al^18^ showed that dopamine transporter at the neuronal membrane of the striatum is decreased in RLS patients. In contrast, Kim et al^19^ found an increase in striatal dopamine transporter in elderly RLS patients. The dopamine transporter has been shown to control dopamine transmission through reuptake of extracellular dopamine into presynaptic neuron. Thus, the increased DAT may decrease the spatial and temporal dynamic of dopamine to bind and activate dopamine D_2_ receptor, which in turn produces motor hyperactivity,^26^ as seen in the ID rat.

In addition to the abnormality in the central nervous system, peripheral neuropathy may also contribute to motor hyperactivity in ID rats. A sensory fiber abnormality has been reported in RLS patients.^27^, ^28^, ^29^, ^30^ Clinical studies showed a high prevalence of RLS in patients with celiac disease^31^ and multiple sclerosis,^32^ who have iron‐deficiency anemia^33^, ^34^ and peripheral neuropathy.^35^, ^36^ Peripheral nerve conduction velocity has been found to be positively correlated with hemoglobin and hematocrit levels.^37^, ^38^ Indeed, ID anemic patients have lower peripheral nerve conduction velocity than normal controls, and this peripheral neuropathy can be normalized by iron supplements.^37^, ^38^ Thus, mechanisms underlying motor hyperactivity caused by iron deficiency and improved PLM in sleep and in wake from iron replacement, which were seen in the present study, may also occur via the peripheral nervous system.

In conclusion, we found that ID rats have signs of RLS, including sleep fragmentation and motor hyperactivity in wake and in sleep. Hematocrit levels and sleep quality were improved and motor hyperactivity was reversed after ID rats were treated with iron. Motor hyperactivity in ID rats can also be corrected by systemic administration of pramipexole. Although the majority of the RLS is not caused by iron‐deficiency anemia, the ID rat can serve as an animal model of ID anemic‐induced RLS. This animal model will allow us to study mechanisms underlying the motor component of ID anemic‐induced RLS, as well as to test potential drugs for the better treatment of ID anemic‐induced RLS.

## Authors' Roles

Yuan‐Yang Lai: conception, organization, and execution of the research project; design and review/critique the statistical analysis; and writing of the first draft of the manuscript.

Yu‐Hsuan Cheng: execution of the research project.

Kung‐Chiao Hsieh: execution of statistical analysis

Darian Nguyan: execution of the research project.

Keng‐Tee Chew: execution of the research project.

Lalini Ramanathan: execution of the research project.

Jerome M. Siegel: conception of research project, review and critique of the manuscript.

## Supporting information

Additional Supporting Information may be found in the online version of this article at the publisher's website.

Supplementary Information

Supplementary Information Polygraphic recording showing PLM in sleep displayed in the video.
