# Supplementary figures and images for: Motor hyperactivity of the iron‐deficient rat — an animal model of restless legs syndrome
Source: Mov Disord. 2017 Aug 26;32(12):1687–93. doi: 10.1002/mds.27133 (PMC5759344; doi:10.1002/mds.27133)

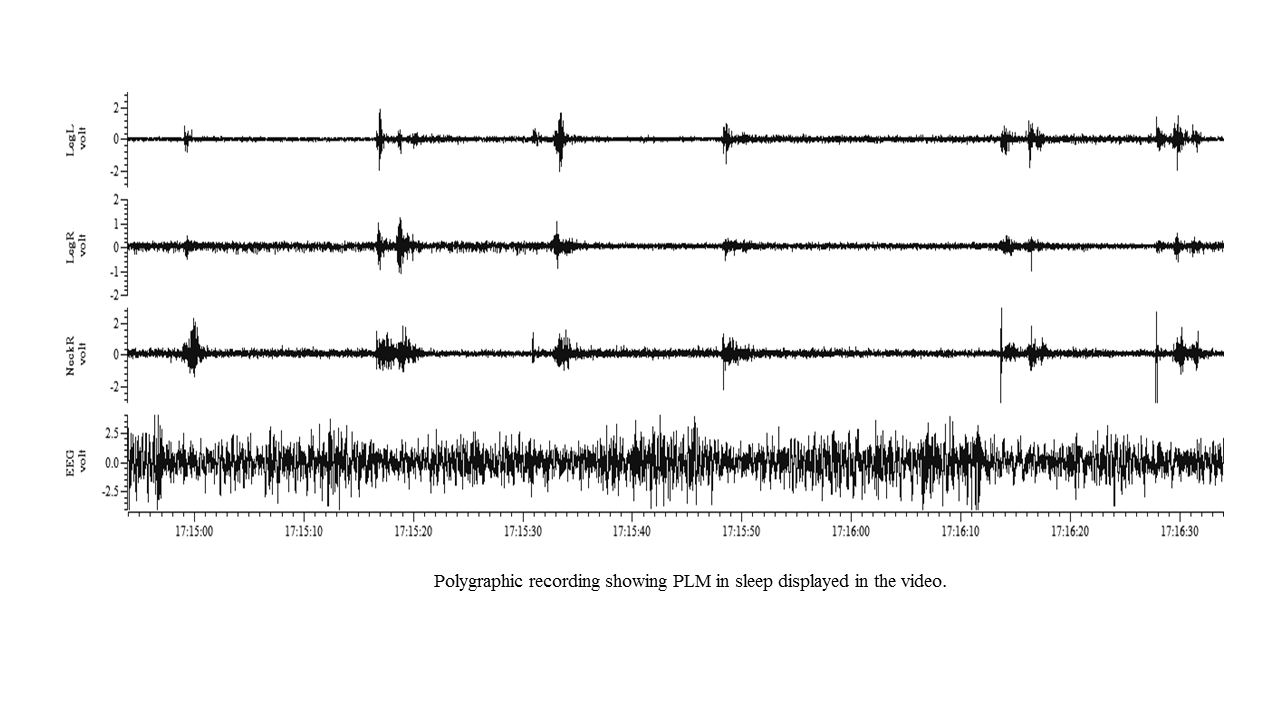

Supplement: Supplementary file 1 — Supplementary Information [file MDS-32-1687-s001.tif]
